# The immune and circulatory systems are functionally integrated across insect evolution

**DOI:** 10.1126/sciadv.abb3164

**Published:** 2020-11-25

**Authors:** Yan Yan, Julián F. Hillyer

**Affiliations:** Department of Biological Sciences, Vanderbilt University, VU Station B 35-1634, Nashville, TN 37235, USA.

## Abstract

The immune and circulatory systems of mammals are functionally integrated, as exemplified by the immune function of the spleen and lymph nodes. Similar functional integration exists in the malaria mosquito, *Anopheles gambiae*, as exemplified by the infection-induced aggregation of hemocytes around the heart valves. Whether this is specific to mosquitoes or a general characteristic of insects remained unknown. We analyzed 68 species from 51 families representing 16 orders and found that infection induces the aggregation of hemocytes and pathogens on the heart of insects from all major branches of the class Insecta. An expanded analysis in the holometabolous mosquito, *Aedes aegypti*, and the hemimetabolous bed bug, *Cimex lectularius*, showed that infection induces the aggregation of phagocytic hemocytes on the hearts of distantly related insects, with aggregations mirroring the patterns of hemolymph flow. Therefore, the functional integration of the immune and circulatory systems is conserved across the insect tree of life.

## Teaser

Infection induces the migration of immune cells to the heart of insects.

## INTRODUCTION

**Fig. 1:**
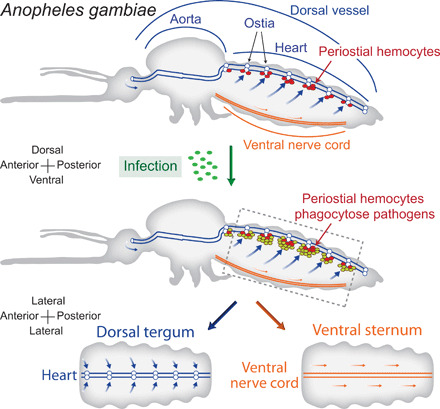
Diagram that illustrates periostial hemocyte aggregation and the experimental design of this study. The top shows a lateral view of an entire mosquito and marks the position of the dorsal vessel (divided into a thoracic aorta and an abdominal heart), periostial hemocytes (red circles) surrounding the ostia (white circles), and the ventral nerve cord. The middle shows that infection induces the aggregation of additional hemocytes (olive green circles) and the phagocytosis of pathogens around the heart’s ostia. The bottom shows a coronal view of the dorsal (tergum) and ventral (sternum) abdomen, which represents how they were visualized and photographed for this study. The arrows mark the direction of hemolymph flow during periods of anterograde heart contractions.

The insect body cavity is a dynamic environment where the insect blood, called hemolymph, constantly and rapidly flows in a manner that bathes all tissues (*1*–*3*). This flow is primarily driven by a dorsal vessel that is structurally divided into an aorta in the thorax and a heart in the abdomen (*4*, *5*). When pathogens invade an adult mosquito and reach its hemocoel, the flow of hemolymph disperses them to all regions of the body (*4*, *6*). Hemolymph flow also circulates immune cells called hemocytes that survey the body for invaders. However, not all hemocytes circulate. Sessile hemocytes exist attached to tissues, yet their distribution is not homogeneous; they concentrate on the outer surface of the dorsal vessel and, specifically, in the regions of the heart that surround the valves, or ostia—locations called the periostial regions (*7*, *8*). Within seconds of infection, these heart-associated hemocytes, called periostial hemocytes, phagocytose circulating pathogens, and soon thereafter, additional hemocytes migrate to the periostial regions and amplify the phagocytosis response (*7*, *9*). Periostial immune responses are advantageous because they occur in areas of high hemolymph flow, placing hemocytes where they are most likely to encounter and destroy pathogens (*9*). Thus, in a manner functionally similar to how the spleen and lymph nodes of vertebrate animals capture pathogens circulating in the blood and lymph (*10*), the function of periostial hemocytes exemplifies the functional integration of the immune and circulatory systems of mosquitoes (Fig. 1).

The biology of periostial hemocytes has only been characterized in the African malaria mosquito, *Anopheles gambiae* (*7*–*9*, *11*, *12*), but hemocytes have also been detected in the lumen of the heart of a stick insect and on the surface of the heart of adult fruit flies and larvae of the greater wax moth (*13*–*17*). Whether the hemocytes of these insects are present near the ostia or whether their response to infection is linked to circulatory currents remained unknown. Hence, we asked whether the functional integration of the immune and circulatory systems is a novel evolutionary trait specific to mosquitoes or a general characteristic of insects. To answer this question, we analyzed 68 species from 51 families representing 16 orders and found that an infection induces the aggregation of hemocytes and pathogens on the heart of insects from all major branches of the class Insecta. Therefore, the functional integration of the immune and circulatory systems is conserved across the insect tree of life.

## RESULTS

### Infection induces the aggregation of phagocytic hemocytes on the heart of holometabolous and hemimetabolous insects

Having observed the interaction between the immune and circulatory systems in the mosquito, *A. gambiae* (Fig. 1), we conducted a comprehensive analysis of infection-induced hemocyte aggregation on the heart of the yellow fever mosquito, *Aedes aegypti*, and the bed bug, *Cimex lectularius*. These two insect pests diverged ~370 million years ago and have different developmental trajectories: One is holometabolous and the other is hemimetabolous (*18*). Moreover, both are societally important; *A. aegypti* transmits human diseases such as dengue and Zika, and *C. lectularius* is a notorious hematophagous pest.

In preparation for studying the functional integration of the immune and circulatory systems of *A. aegypti* and *C. lectularius*, we quantified how efficiently their hemocytes could be labeled by injecting Vybrant CM-DiI into the hemocoel and examining their perfused hemocytes 20 to 30 min later (fig. S1). Vybrant CM-DiI is a lipophilic dye that, in *A. gambiae*, labels the circulating and sessile hemocytes but does not label the heart, pericardial cells, integument, or any other tissue (*7*–*9*). Moreover, this dye has also been used to label the hemocytes of *A. aegypti* and *Apis mellifera* (Hymenoptera) (*19*, *20*), and therefore, we hypothesized that it could label the hemocytes of any insect. We found that CM-DiI efficiently stains the hemocytes of naïve, injured, and *Escherichia coli*–infected mosquitoes and bed bugs. On average, 84, 83, and 77% of the hemocytes from naïve, injured, and *E. coli*–infected *A. aegypti*, respectively, were stained with CM-DiI. Similarly, 84, 90, and 89% of the hemocytes from naïve, injured, and *E. coli*–infected *C. lectularius*, respectively, were stained with CM-DiI. Fat body and other cells were seldomly stained with CM-DiI, similar to what we have observed for *A. gambiae* (*7*–*9*, *21*).

**Fig. 2:**
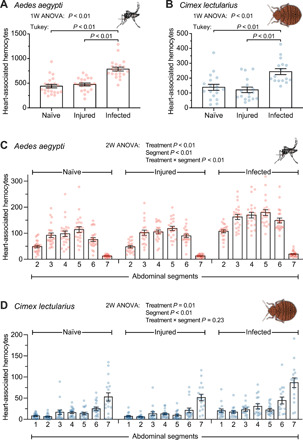
Infection induces the aggregation of phagocytic hemocytes on the heart of *A. aegypti* and *C. lectularius*. (**A** and **B**) Hemocytes on the heart of naïve, injured, and *E. coli–*infected *A. aegypti* (A) and *C. lectularius* (B). 1W ANOVA, one-way analysis of variance. (**C** and **D**) Spatial distribution of hemocytes along the heart in the different abdominal segments of naïve, injured, and *E. coli–*infected *A. aegypti* (C) and *C. lectularius* (D). Column heights mark the mean, and the whiskers denote the SEM. Each circle represents the number of heart-associated hemocytes in an individual insect. 2W, two-way.

We then assayed for the presence of hemocytes on the heart of mosquitoes and bed bugs by injecting CM-DiI into the hemocoel, bisecting their abdomen, and examining the tubular heart that extends across the dorsal tergum. For *A. aegypti*, approximately 440 hemocytes reside on the heart of a naïve mosquito (Fig. 2A). Injury does not alter the number of periostial hemocytes, but infection results in a 1.7-fold increase in the number of periostial hemocytes. This indicates that, much like occurs in adult *A. gambiae* (*7*), an infection induces the recruitment of additional hemocytes to the heart. A more detailed analysis of the spatial distribution of hemocytes revealed that most hemocytes aggregate in the periostial regions of abdominal segments 3 to 6 (Fig. 2C). Again, this aggregation pattern resembles that of *A. gambiae*, which is advantageous because these middle abdominal segments are the locations that have the swiftest hemolymph flow (*9*). In bed bugs, we observed similar results. Specifically, the average naïve and injured bed bug has 140 and 120 hemocytes on the heart, respectively, but infection induces a twofold increase in the number of heart-associated hemocytes (Fig. 2B). In *C. lectularius*, hemocytes predominantly aggregate in the portions of the heart of abdominal segments 6 and 7 (Fig. 2D). This spatial distribution occurs because this portion of the heart is enlarged and is where the incurrent ostia are located, as evidenced by structural analyses of the heart of other hemipterans, such as the kissing bug, *Rhodnius prolixus* (*22*), and the boxelder bug, *Leptocoris trivittatus* (*23*).

**Fig. 3:**
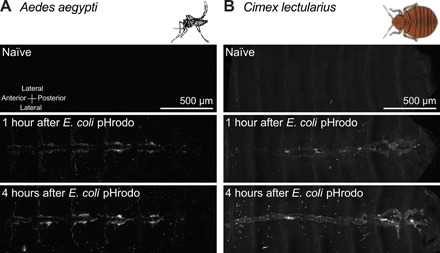
Heart-associated hemocytes phagocytose bacteria in *A. aegypti* and *C. lectularius*. (**A** and **B**) Phagocytosis of *E. coli* pHrodo by the hemocytes of *A. aegypti* (A) and *C. lectularius* (B). Insects were imaged before injection (naïve; negative control) or at 1 and 4 hours after injection with *E. coli* pHrodo. Fluorescence images show the entire length of the dorsal abdomen of each insect, with the heart extending along the horizontal midline. The heart-associated hemocytes, as well as other sessile hemocytes dispersed throughout the abdomen, actively phagocytose pathogens that circulate with the hemolymph.

To determine whether the hemocytes that aggregate on the heart are immunologically active, we injected *A. aegypti* and *C. lectularius* with *E. coli* bioparticles conjugated to pHrodo, which is a pH-sensitive dye that only fluoresces in an acidic environment, such as that of the phagolysosome. Therefore, this dye is an efficient marker for phagocytosis (*9*). In naïve mosquitoes and bed bugs, no fluorescence was detected, which was expected because no *E. coli* pHrodo was injected. However, when mosquitoes and bed bugs were injected with *E. coli* pHrodo, we detected fluorescence emission soon after injection, and this fluorescence was predominantly in the areas that contain the heart-associated hemocytes (Fig. 3, A and B). Together, these data show that, in both holometabolous and hemimetabolous insects, infection induces the aggregation of hemocytes on the heart and that these hemocytes rapidly phagocytose pathogens that circulate with the hemolymph.

### Hemocytes and pathogens aggregate on the hearts of taxonomically diverse insects

**Fig. 4:**
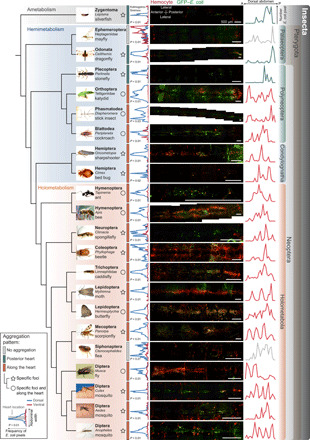
The heart-associated immune response is a trait shared across the insect tree of life. On the left is a selection of the insects assayed, arranged by insect phylogeny. The fluorescence images near the center show the entire length of the dorsal abdomen of each insect, with the heart extending along the horizontal midline. They show that hemocytes (red) and GFP–*E. coli* (green) aggregate and colocalize on the heart, although more than one pattern was observed (see box for key). To the immediate left of the images are frequency distributions of GFP–*E. coli*–positive pixels along the lateral axis of the dorsal (blue lines) and ventral (red lines) abdomens. To the immediate right of the images are frequency distributions of GFP–*E. coli*–positive pixels along the anterior-posterior axis of the dorsal abdomen. The data show that, except in the mayfly and flea, pathogens aggregate on the heart (blue peaks in the center of the leftmost graphs) and nowhere else. Moreover, peaks in the rightmost graphs show that hemocytes aggregate in the periostial regions along the length of the heart, except in silverfish, dragonflies, and stoneflies, where they aggregate on the periostial regions of the posterior of the heart.

Given that periostial hemocyte aggregation occurs in both holometabolous mosquitoes and hemimetabolous bed bugs, we next sought to assess whether periostial immune responses occur throughout the class Insecta. We initiated this comprehensive survey by infecting field-collected *Anopheles punctipennis* (Diptera: Anophelinae), *Aedes albopictus* (Diptera: Culicinae), and *Culex* sp. (Diptera: Culicinae) with green fluorescent protein (GFP)–expressing *E. coli* to induce the hemocyte aggregation response. Following hemocyte labeling with CM-DiI, we bisected the mosquito’s abdomen and visualized the distribution of hemocytes and pathogens on (i) the tubular heart that extends across the dorsal tergum and (ii) the ventral nerve cord that extends across the ventral sternum (Fig. 1). Both the dorsal and ventral sides of the abdomen were examined because the ventral nerve cord mirrors the location of the heart but is not in a region of high hemolymph flow (*24*). Therefore, if an interaction between the immune and circulatory systems was to exist, hemocytes and pathogens would aggregate on the heart but not on the ventral nerve cord. Much like we found in our *A. gambiae* laboratory colony, in both anopheline and culicine mosquitoes, hemocytes and pathogens aggregate exclusively around the six pairs of cardiac ostia and nowhere else in the tergum or sternum (Fig. 4 and fig. S2).

We next used the same approach to examining members of Pterygota within Holometabola (synonym Endopterygota). In 7 species in Diptera, 2 in Mecoptera, 11 in Lepidoptera, 4 in Trichoptera, 9 in Coleoptera, 1 in Neuroptera, and 6 in Hymenoptera, we once again found that hemocytes and pathogens aggregate along the entire length of the heart in the dorsal abdomen and nowhere else in the body (Fig. 4). Closer examination of the distribution of hemocytes and GFP–*E. coli* revealed two different patterns, but both included hemocyte aggregation around the ostia (Fig. 4 and figs. S2 to S7). In the first pattern, observed in scorpionflies (Mecoptera: Panorpidae), moths (Lepidoptera: Noctuidae), beetles (Coleoptera: Scarabaeidae), and spongillaflies (Neuroptera: Sisyridae), hemocytes and pathogens aggregate in specific foci on the surface of the heart in a manner that is similar to what occurs in the periostial regions of mosquitoes. In the second pattern, observed in house flies (Diptera: Muscidae), butterflies (Lepidoptera: Nymphalidae), caddisflies (Trichoptera: Limnephilidae), honeybees (Hymenoptera: Apidae), and ants (Hymenoptera: Formicidae), hemocytes and pathogens concentrate in specific foci, but they are also sparsely distributed between some of the foci. We failed to detect heart-associated immune responses in the cat flea (Siphonaptera: Pulicidae), where similar amounts of pathogens were present on the dorsal and ventral abdomen. We hypothesize that this is due to variation in circulatory physiology that is associated with the flea’s laterally flattened body shape.

Once we found that heart-associated immune responses occur throughout Holometabola, we investigated hemimetabolous species in Neoptera. In six species in Hemiptera, three in Blattodea, one in Phasmatodea, seven in Orthoptera, and two in Plecoptera, we confirmed that hemocyte aggregation only occurs in cardiac tissues and nowhere else in the body (Fig. 4 and figs. S8 to S10). Within Condylognatha, hemocytes and pathogens aggregate in specific foci on the heart of bed bugs (Hemiptera: Cimicidae) and sharpshooters (Hemiptera: Cicadellidae). Moreover, within Polyneoptera, hemocytes and pathogens are both in foci and sparsely distributed between some foci in cockroaches (Blattodea: Blattidae), walking sticks (Phasmatodea: Diapheromeridae), and katydids (Orthoptera: Tettigoniidae). The pattern seen in these Polyneoptera could be because their elaborate dorsal diaphragm provides a larger and more continuous platform for the aggregation of hemocytes (*25*). A completely different pattern occurs in one Polyneoptera—the stonefly (Plecoptera: Perlidae)—where hemocytes and pathogens aggregate only in the heart regions located in the posterior abdominal segments. Although plecopterans have segmental ostia (*25*), it is possible that their distinct pattern of hemocyte aggregation occurs because only the posterior ostia are functional. An alternative explanation is that a reduced dorsal diaphragm reduces the ability of hemocytes to adhere to the heart (*26*).

We then examined another hemimetabolous group: the Paleoptera. In two dragonfly species (Odonata: Libellulidae), hemocytes and pathogens aggregate near the posterior of the heart in a manner that resembles the aggregation pattern in mosquito larvae (Fig. 4 and fig. S10) (*27*). This makes sense given the parallels in circulatory physiology between dragonfly adults and mosquito larvae; odonate adults only have two pairs of abdominal ostia that are located in the posterior of the abdomen, which is similar to how mosquito larvae only allow hemolymph to enter the heart via a posterior incurrent opening (*2*, *25*). Therefore, it appears that their circulatory physiology drives hemocytes and pathogens only to the posterior of the abdomen. A completely different pattern was observed in two mayfly species (Ephemeroptera: Heptageniidae); few hemocytes and pathogens are attached to the abdominal integument, with slightly more hemocytes in the ventral abdomen than in the dorsal abdomen (Fig. 4 and fig. S10). This suggests that heart-associated immune responses do not occur in Ephemeroptera, although ostia are present in all or most abdominal segments (*28*). Because mayfly adults only live ~2 days, we hypothesize that these nonfeeding and short-lived adult insects minimize their investment in immunity in favor of reproduction.

Last, we examined wingless insects that do not undergo metamorphosis (ametabolous) and are the sister group to the Pterygota. Excitingly, infection of silverfish (Zygentoma: Lepismatidae) results in both hemocytes and pathogens distinctively aggregating within the periostial regions—especially toward the posterior end of the heart—although the strength of hemocyte aggregation is less pronounced when compared to more derived insect groups (Fig. 4 and fig. S10). The pattern observed in silverfish mirrors the pattern observed in odonates and plecopterans, raising the possibility that infection-induced hemocyte aggregation at the posterior of the heart is the pleisiomorphic state. Together, these data show that the immune and circulatory systems are functionally integrated throughout the insect lineage.

## DISCUSSION

Substantial efforts have been made to characterize the immunological mechanisms used by insects to fight infection (*29*), yet less attention has been paid to the structural features and functional mechanics of hemolymph propulsion (*4*). Moreover, until recently, how circulatory currents affect immune responses has gone ignored (*4*). This is unexpected because the immune responses of vertebrate animals are intrinsically linked to the flow of blood and lymph (*10*). To address this gap in knowledge, we conducted a comprehensive survey in the class Insecta and, here, show that immunologically active hemocytes are present on the hearts of holometabolous, hemimetabolous, and ametabolous insects and that an infection induces the migration of hemocytes to the periostial regions of the heart, therefore amplifying the immune response.

Although this study uncovered the physiological interaction between two major organ systems, the mechanisms governing this interaction remain mostly unknown. Thioester-containing complement-like proteins and Nimrod family proteins are immune factors that influence the migration of hemocytes to the heart of mosquitoes and fruit flies (*11*, *12*, *16*, *30*). Both of these protein families are encoded in the genomes of diverse insects (*31*, *32*), so their roles in heart-associated responses likely extend beyond Diptera. In addition, a collagen protein that is part of the cardiac extracellular matrix, called Pericardin, facilitates the aggregation of hemocytes on the heart of fruit flies (*13*). Collectively, this means that hemocyte migration to the heart is driven by a combination of immune and cardiac components.

The directional forces of circulatory currents undoubtedly facilitate how hemocytes migrate to the heart. In mosquitoes, hemocytes aggregate in the periostial regions of abdominal segments 2 to 7 and, more precisely, in the locations of the heart that contain the incurrent ostia. Most of these hemocytes aggregate in the periostial regions of the middle abdominal segments, which are the locations of the ostia that receive the most hemolymph flow (*9*). In a similar circulatory pattern, the hemocytes of dragonflies and silverfish aggregate on the posterior of the heart, which is where their incurrent ostia are located (*25*). Given that hemocytes aggregate in areas of high hemolymph flow, it makes sense that allatotropin, which is a neuropeptide that modulates heart rhythmicity (*33*), also alters the number of hemocytes present on the surface of the heart (*19*). In addition, linking immunity and circulation are nitric oxide and lysozymes. They are produced by hemocytes—including periostial hemocytes—to combat bacterial infections, but they also decelerate the insect heart contraction rate (*14*, *29*, *34*, *35*). Nitric oxide also has immune and circulatory functions in vertebrate animals (*36*, *37*). Therefore, the molecular drivers of the physiological interaction between the immune and circulatory systems are undoubtedly complex but are likely conserved across the insect lineage and beyond.

From an evolutionary perspective, insects are hexapods that are nested within a paraphyletic Crustacea, which, collectively, is called the Pancrustacea (*38*). Innovation in the hexapod lineage resulted in the evolution of the tracheal system and the decoupling of hemolymph circulation and gas exchange, which led to a decrease in vasculature and a simplification of the major circulatory organs (*4*, *39*). This simplification resulted in a dorsal vessel that contains ostia and propels hemolymph in three primary ways: (i) bidirectional flow as occurs in Diplura (a noninsect Hexapod) and wingless ametabolous insects, (ii) anterograde flow as occurs in hemimetabolous insects, and (iii) periodic alternation between anterograde and retrograde flow as occurs in holometabolous insects (*4*, *5*). To our knowledge, no studies have investigated how noninsect hexapods (Protura, Collembola, and Diplura) immunologically respond to infection. Regardless, there are many similarities in the immune and circulatory systems of insects and crustaceans (*40*). For example, the primary immune cells in both insects and crustaceans are hemocytes, and the major immune effector pathways are conserved between these two groups (*40*). Moreover, insects and crustaceans both have open circulatory systems that are composed of a hemocoel, hemolymph, and a heart that is located along the dorsal midline (*4*, *5*). Many of the same neuropeptides (e.g., crustacean cardioactive peptide and FMRFamide-like peptides) and neurotransmitters (e.g., serotonin and octopamine) influence cardiac physiology in both animal groups (*4*). Given all these parallels, we hypothesize that the interaction between the circulatory and immune systems extends beyond insects and into noninsect hexapods and crustaceans. Although differences in the architecture of the circulatory systems of insects and crustaceans preclude a direct structural comparison, hemocytes populate the endothelium of the hepatic arterioles of lobsters (*41*), and following an infection, they aggregate on the heart and arterial vessels of prawns and crabs (*42*, *43*). In penaeid shrimp and prawns, heart contractions drive hemolymph into a lymphoid organ, where immune cells destroy circulating pathogens and release humoral immune factors into circulation (*44*, *45*). Therefore, hemocytes in the circulatory structures of decapod crustaceans function in a manner reminiscent of the periostial hemocytes of insects.

In conclusion, insects emerged ~480 million years ago, and Zygentoma diverged from Pterygota ~420 million years ago (*18*). The data presented herein show the conserved association of hemocytes and immune responses on the heart of species that span the entire insect lineage. Therefore, the functional integration of the circulatory and immune systems of insects likely evolved near the origin of the insect lineage or predates the divergence of Insecta from other Pancrustacea.

## MATERIALS AND METHODS

### *A. aegypti* and *C. lectularius* colonies

*A. aegypti* Black Eye Liverpool strain was obtained from the BEI Resources (catalog no. NR-48921, Manassas, VA). Mosquitoes were maintained at 27°C and 75% relative humidity under a 12-hour:12-hour light:dark photoperiod. Adults were maintained in 2.4-liter plastic buckets and fed 10% sucrose. Five-day-old female mosquitoes were used in the experiments.

*C. lectularius* were obtained from a colony maintained at the Purdue University. Bed bugs were starved for 7 days or more at room temperature before experimental manipulations. A mixture of male and female adult bed bugs of unknown age was used.

### Surveyed insects, identification, and phylogeny

Insects were collected in the wild using a sweep net or a light trap or were obtained from established laboratory colonies. Insects were identified to the family or genus level by their external morphology (table S1), and insect phylogeny was inferred from Misof *et al.* (*18*). The following sources were used in the identification of insects: (i) Kaufman Field Guide to Insects of North America (*46*), (ii) bugguide.net, (iii) and the artificial intelligence model powered by iNaturalist or Seek apps. When identifying insects, consideration was given to their ecology, including geographic distribution, collection site, and time of year. Table S1 details the insects used in this study, including the location and date of collection, the collectors, the infection doses, and other relevant information. Collecting done in state parks or state natural areas was performed pursuant to the State of Tennessee, Department of Environment and Conservation, Division of Natural Areas Scientific Study permit no.: 2019-017. From the time of collection to the time of experimentation, insects were fed a 10% sucrose solution and maintained in a BugDorm (MegaView Science Co., Taiwan) under standard laboratory conditions.

### Bacterial growth and insect infection

Tetracycline-resistant, GFP-expressing *E. coli* was grown overnight in Luria-Bertani’s (LB) rich nutrient medium in a 37°C shaking incubator (New Brunswick Scientific, Edison, NJ, USA). The absorbance of GFP–*E. coli* cultures was measured spectrophotometrically and normalized to an optical density at 600 nm of 5 before injection. To initiate infections, insects were briefly anesthetized in a tube or Petri dish held over ice and then intrathoracically injected using either a Nanoject III Programmable Nanoliter Injector (Drummond Scientific Company, Broomall, PA, USA) when the injected volume was <2 μl or a calibrated micropipette (Drummond Scientific Company, Broomall, PA, USA) when the injected volume was >2 μl. The injected volume for each insect was normalized to approximately 69 nl per 1 mg of insect weight. The absolute number of *E. coli* injected into each insect was calculated after plating dilutions of the tetracycline-resistant, GFP–*E. coli* culture on an LB plate containing tetracycline and counting the resultant colony-forming units.

### *A. aegypti* and *C. lectularius* CM-DiI hemocyte staining efficiency

Mosquitoes were left unmanipulated (here termed naïve), injured by injecting 69 nl of LB medium or infected by injecting 69 nl of GFP–*E. coli*. One hour later, each mosquito was injected in the thorax with a solution of 67 μM CM-DiI Cell-Labeling Solution (Thermo Fisher Scientific, Waltham, MA, USA) and 1.08 mM Hoechst 33342 (Thermo Fisher Scientific) in phosphate-buffered saline (PBS) until its abdomen became expanded. This protocol specifically labels circulating and sessile hemocytes with CM-DiI and all cell nuclei with Hoechst 33342 (*7*). It was crucial that the staining solution was injected within minutes of its preparation because once the CM-DiI is placed in an aqueous environment, its hemocyte staining effectiveness begins to decrease, approaching 0% after 10 to 15 min of mixing (*7*). At 20 to 30 min later, the hemolymph with circulating hemocytes was perfused by making a small incision at the ventral side of the seventh abdominal segment and then injecting PBS through the thoracic anepisternal cleft. The first five drops of hemolymph that exited the abdomen were collected within a 1-cm-diameter etched ring on a glass slide. The circulating hemocytes were allowed to adhere to the slide for 20 min in a humidity chamber, fixed for 5 min by adding 4% formaldehyde in PBS, and washed three times with PBS for 5 min each, and a coverslip was mounted using Aqua-Poly/Mount (Polysciences, Warrington, PA, USA). A similar protocol was followed for bed bugs, except that the hemolymph was perfused by making a small incision between the sixth and seventh abdominal segments, and PBS was injected through the ventral thorax.

Hemocyte staining efficiency for each insect was measured by examining the first 50 hemocytes that were viewed by simultaneous differential interference contrast (DIC) and fluorescence microscopy on a Nikon 90i compound microscope connected to a Nikon Digital Sight DS-Qi1 monochrome digital camera and Nikon’s Advanced Research NIS-Elements software (Nikon, Tokyo, Japan). Cells were considered hemocytes if they had both a nucleus (stained with Hoechst 33342 and seen in the blue channel) and a cell membrane (seen in the DIC channel). Then, hemocytes were considered stained if they had incorporated CM-DiI (seen in the red channel). Hemocytes were distinguished from fat body cells by their substantially smaller size and the absence of large, refractive lipid droplets. Hemocytes were distinguished from the nuclei of lysed cells by examining the DIC channel; nuclei from lysed cells lack a cell membrane. Three independent trials were performed for both *A. aegypti* and *C. lectularius*. Combined, at least 24 mosquitoes and 15 bed bugs were analyzed per treatment group, respectively. Data were analyzed by one-way analysis of variance (ANOVA), followed by Tukey’s multiple comparison test (GraphPad Prism, San Diego, CA).

### In vivo hemocyte staining and dissection of the dorsal and ventral abdomen

For all insects used in this study, at 1 or 4 hours following infection, hemocytes were stained in vivo using Vybrant CM-DiI as described above. Then, each insect was fixed for 10 min by injecting 16% formaldehyde into the hemocoel until the abdomen began to expand. The head and thorax of each insect were separated from the abdomen using a razor blade, and for insects collected in the wild, the head and thorax were stored in denatured ethanol at −20°C in case further identification was required. The abdomen was then bisected along a coronal plane and immersed in PBS containing 0.1% Triton X-100, and the internal organs were removed. The dorsal abdomen (containing the heart) and the ventral abdomen (containing the ventral nerve cord) were rinsed briefly in PBS and mounted between a glass slide and a coverslip using Aqua-Poly/Mount. Note that some insects were processed at 1 hour after infection, whereas others were processed at 4 hours after infection. For species that were processed at both time points, the results were similar, except that stronger aggregations were sometimes seen at 4 hours.

### Visualization and quantification of hemocyte aggregation on the heart of *A. aegypti* and *C. lectularius*

The dissected dorsal abdomens of naïve, injured, and GFP–*E. coli*–infected *A. aegypti* and *C. lectularius* were imaged under bright-field and fluorescence illumination. Z-stacks were acquired using a linear encoded Z-motor, and for image presentation, all images within a stack were combined into a two-dimensional, focused image using the extended depth of focus (EDF) function in NIS-Elements.

The heart-associated hemocytes were counted manually by examining all images within a Z-stack. A cell was counted as a heart-associated hemocyte if it resided near the dorsal vessel and was labeled with both CM-DiI and Hoechst 33342. The heart-associated hemocytes were counted in abdominal segments 2 to 7 in *A. aegypti* and 1 to 7 in *C. lectularius*. The heart-associated hemocytes in segment 1 of *A. aegypti* were not counted because this is the location of the thoracoabdominal ostia. This region is structurally conserved across the dipteran lineage, and its circulatory physiology is different from the other abdominal segments and is a location where few hemocytes are located (*21*, *47*). Three independent trials were performed for both *A. aegypti* and *C. lectularius*. Combined, at least 21 mosquitoes and 16 bed bugs were analyzed per treatment group, respectively. Data were analyzed by one-way ANOVA, followed by Tukey’s multiple comparison test.

### Visualization of the phagocytic activity of heart-associated hemocytes in *A. aegypti* and *C. lectularius*

*E. coli* bacterial bioparticles conjugated to pHrodo Red (Thermo Fisher Scientific) were reconstituted in PBS at 2 mg/ml. *A. aegypti* and *C. lectularius* were injected with 0.4 and 1 μl of pHrodo Red *E. coli*, respectively. At 1 and 4 hours after challenge, each insect was injected with 16% formaldehyde, and the dorsal abdomen was dissected and mounted as described above. Insects that were not injected were used as negative controls. Each dorsal abdomen was visualized under bright-field and fluorescence illumination, and images were acquired as detailed above. All images within a Z-stack were combined into a focused image using the EDF function in NIS-Elements, and the pHrodo Red channel was exported in monochrome. This experiment was replicated in three to four insects per treatment group for each species.

### Visualization and quantification of hemocytes and pathogens in surveyed insects

Each dissected dorsal and ventral abdomen from an infected insect was imaged under bright-field and fluorescence illumination as described above. Each side of the abdomen was first imaged under low magnification to examine the distribution of hemocytes and GFP–*E. coli* over the entire length of the heart or the ventral nerve cord. Then, a region of the heart—and specifically, a periostial region where the ostia were clearly visible—was examined under high magnification to more clearly visualize the aggregation pattern of both hemocytes and GFP–*E. coli*. When an abdomen was too long to fit in a single frame at the lowest magnification, multiple images along the abdomen were acquired, and the images were stitched together using Adobe Photoshop CC 2019 (San Jose, CA, USA).

The aggregation pattern of hemocytes and pathogens was determined by examining the overlay of three fluorescence channels—red for hemocytes, green for GFP–*E. coli*, and blue for cell nuclei—relative to the position of the heart, as identified in the Z-stacks by bright-field imaging and the cell nuclei fluorescence channel. The judgment of where immune responses occur was based primarily on the GFP–*E. coli* channel because hemocyte staining in insects collected in the wild is noisier and less efficient than in mosquitoes reared in our laboratory. For quantitative analysis of the distribution of GFP–*E. coli*, ImageJ was used to count the pixels that contained GFP–*E. coli* signal in EDF images of the entire dorsal and ventral abdomen (Fig. 4 and figs. S2 to S10). These pixels were defined as the pixels with intensities above the threshold that distinguished GFP emitted by *E. coli* from background fluorescence. Quantitative analyses measured two different types of fluorescence distribution. To create the graphs to the left of the fluorescence images in Fig. 4, images were collapsed along the insect’s anterior-posterior axis such that the number of pixels within a horizontal row that had fluorescence intensity values above the threshold was counted, and the frequency of GFP–*E. coli* pixels was plotted along the width (laterally, from side to side) of the dorsal (blue line) and ventral (red line) abdomen, with the heart and ventral nerve cord on the horizontal midline of each graph. This informs about (i) the relative distribution of fluorescence in the dorsal and ventral sides and (ii) whether fluorescence is concentrated on the heart (blue line with peak in the center) or is evenly distributed throughout the abdomen (blue line with no peak in the center). The frequency distribution of *E. coli* in the dorsal and ventral abdomen was compared by two-sample Kolmogorov-Smirnov test in the R software. To create the graphs to the right of the fluorescence images in Fig. 4, images were collapsed along the insect’s left-right (lateral) axis such that the number of pixels within a vertical column that had fluorescence intensity values above the threshold was counted, and the frequency of GFP–*E. coli* was plotted along the length of the dorsal abdomen, with the anterior of the abdomen on the left and the posterior on the right. Together with the leftmost graphs showing heart-associated aggregation, the rightmost graphs in Fig. 4 inform about whether the GFP–*E. coli* does not aggregate or aggregates (i) in foci at the periostial regions (vertical peaks with valleys), (ii) in both foci and also along the length of the heart (vertical peaks but no consistent valleys), or (iii) at the posterior of the heart (peaks only on the right). Last, the pictures of the whole insects shown in Fig. 4 were either taken by the authors or acquired from the public domain.

## Supplementary Material

http://advances.sciencemag.org/cgi/content/full/6/48/eabb3164/DC1

Adobe PDF - abb3164_SM.pdf

The immune and circulatory systems are functionally integrated acros insect evolution

## SUPPLEMENTARY MATERIALS

Supplementary material for this article is available at http://advances.sciencemag.org/cgi/content/full/6/48/eabb3164/DC1

## Appendix

View/request a protocol for this paper from *Bio-protocol*.
