# Supplementary material for: The immune and circulatory systems are functionally integrated across insect evolution
Source: Sci Adv. 2020 Nov 25;6(48):eabb3164. doi: 10.1126/sciadv.abb3164 (PMC7688319; doi:10.1126/sciadv.abb3164)
Supplement: http://advances.sciencemag.org/cgi/content/full/6/48/eabb3164/DC1 [file supp_6_48_eabb3164__index.html]

Science Advances | Science AdvancesAAASSearchScience AdvancesMenu

## Supplementary Materials

# The immune and circulatory systems are functionally integrated across insect evolution

Yan Yan and Julián F. Hillyer

Download Supplement

**This PDF file includes:**

- Figs. S1 to S10
- Table S1

**Files in this Data Supplement:**

- Adobe PDF - abb3164\_SM.pdf
