# Supplementary material for: The immune and circulatory systems are functionally integrated across insect evolution
Source: Sci Adv. 2020 Nov 25;6(48):eabb3164. doi: 10.1126/sciadv.abb3164 (PMC7688319; doi:10.1126/sciadv.abb3164)
Supplement: http://advances.sciencemag.org/cgi/content/full/6/48/eabb3164/DC1 [file supp_6_48_eabb3164__1.pdf]

Science Advances
AAAS

advances.sciencemag.org/cgi/content/full/6/48/eabb3164/DC1

Supplementary Materials for

The immune and circulatory systems are functionally integrated across insect evolution

Yan Yan and Julián F. Hillyer*

*Corresponding author. Email: julian.hillyer@vanderbilt.edu

Published 25 November 2020, *Sci. Adv.* **6**, eabb3164 (2020)
DOI: 10.1126/sciadv.abb3164

This PDF file includes:

Figs. S1 to S10
Table S1

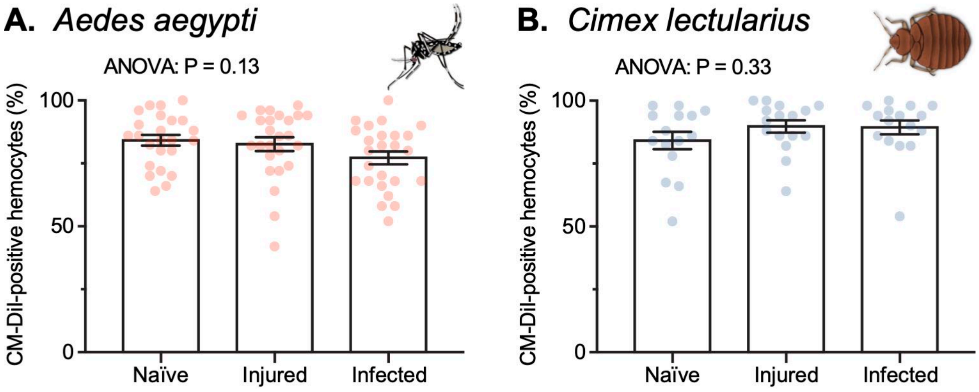

Fig. S1. The efficiency of Vybrant CM-DiI staining of hemocytes in *Aedes aegypti* and *Cimex lectularius*. Quantitative analysis of *in vivo* CM-DiI staining in perfused hemocytes from naïve, injured (LB) and *E. coli*-infected *Ae. aegypti* (**A**) and *C. lectularius* (**B**). Column heights mark the mean and the whiskers denote the standard error of the mean. Each circle represents the percentage of hemocytes that are positively stained by CM-DiI in an individual insect. The vast majority of hemocytes stain with CM-DiI.

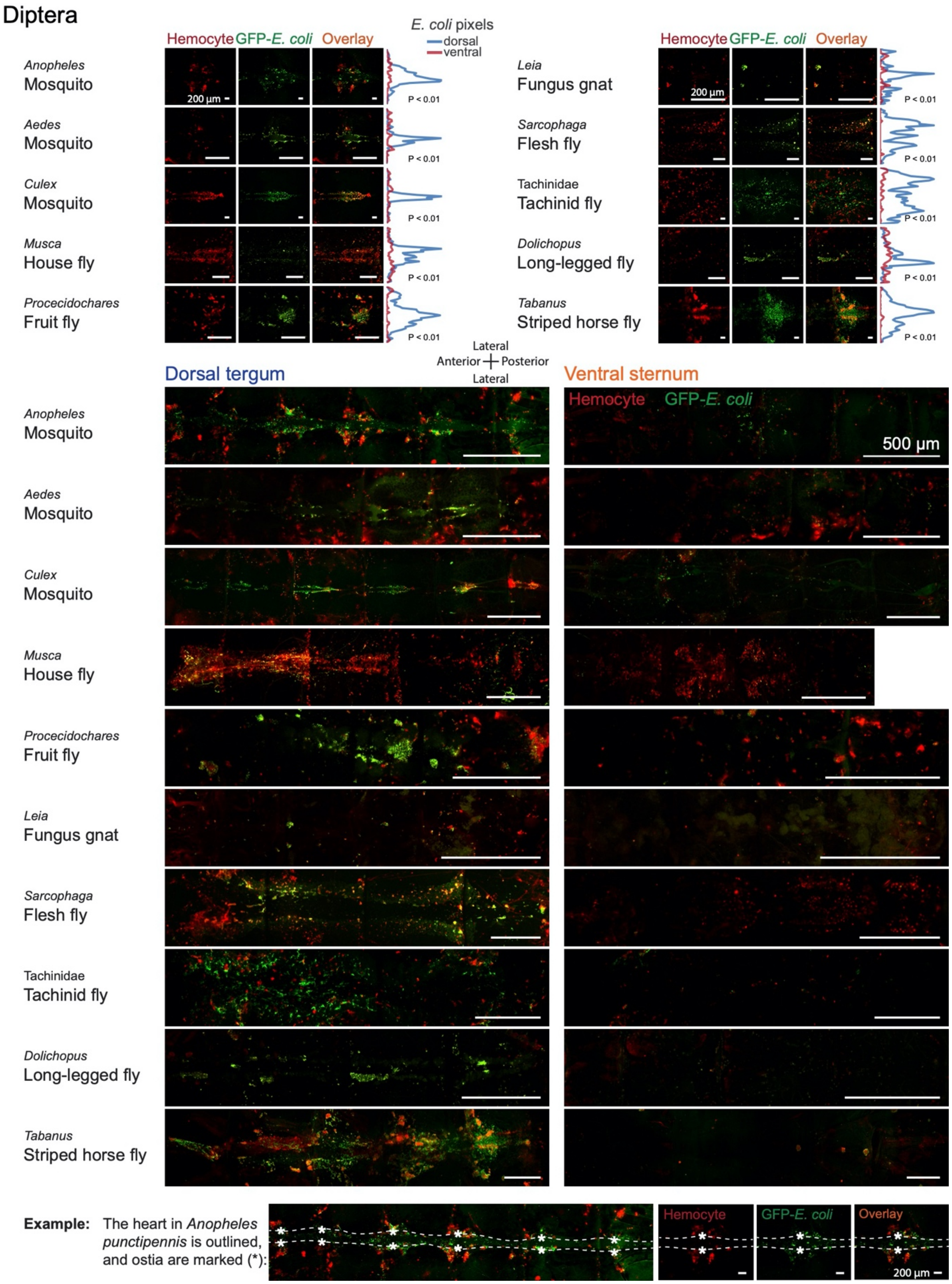

Fig. S2. The aggregation of hemocytes and pathogens on the heart of members of the order Diptera. Fluorescence microscopy images show one region of the heart (top section of the figure) and the entire dorsal and ventral abdomen (bottom section of the figure) in each insect. Graphs show quantification of GFP-*E. coli* pixel frequency along the width of the entire dorsal (blue line) and ventral (red line) abdomen. Hemocytes (red) and GFP-*E. coli* (green) distinctively aggregate and co-localize (overlay) on the heart – but not the surrounding tergum – and are largely absent in the ventral sternum.

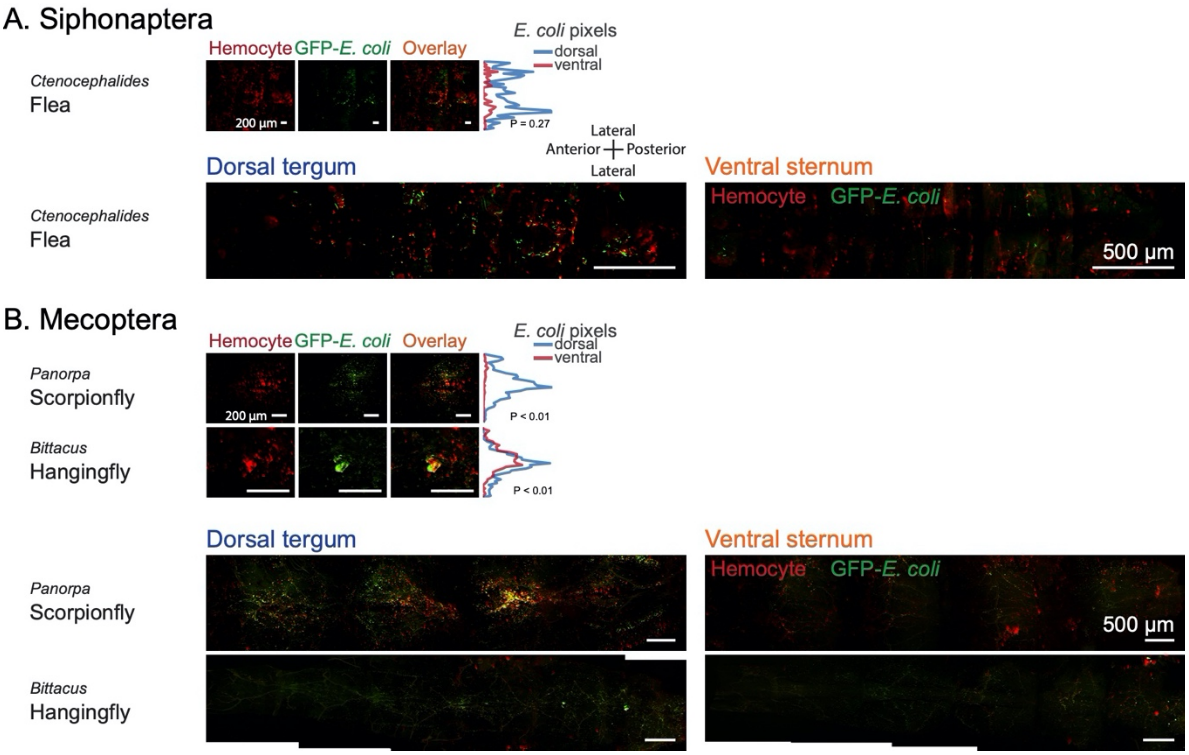

Fig. S3. The aggregation of hemocytes and pathogens on the heart of members of the orders Siphonaptera and Mecoptera. Fluorescence microscopy images show one region of the heart (top section of each panel) and the entire dorsal and ventral abdomen (bottom section of each panel) in each insect. Graphs show quantification of GFP-*E. coli* pixel frequency along the width of the entire dorsal (blue line) and ventral (red line) abdomen. In Siphonaptera (**A**), hemocytes (red) and GFP-*E. coli* (green) do not aggregate or co-localize (overlay) on the heart. In Mecoptera (**B**), hemocytes and GFP-*E. coli* distinctively aggregate and co-localize on the heart – but not the surrounding tergum – and are largely absent in the ventral sternum.

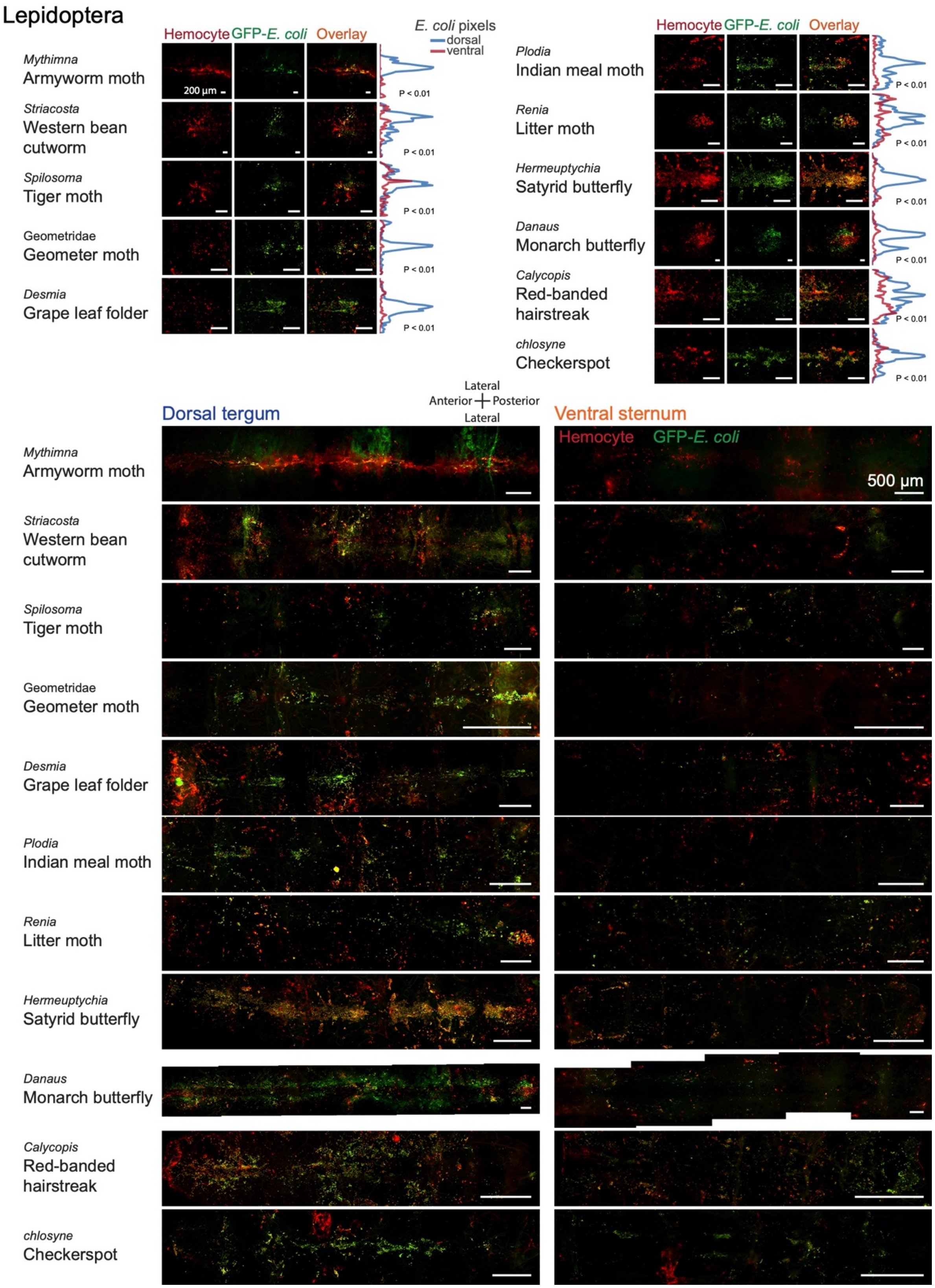

Fig. S4. The aggregation of hemocytes and pathogens on the heart of members of the order Lepidoptera. Fluorescence microscopy images show one region of the heart (top section of the figure) and the entire dorsal and ventral abdomen (bottom section of the figure) in each insect. Graphs show quantification of GFP-*E. coli* pixel frequency along the width of the entire dorsal (blue line) and ventral (red line) abdomen. Hemocytes (red) and GFP-*E. coli* (green) distinctively

aggregate and co-localize (overlay) on the heart – but not the surrounding tergum – and are largely absent in the ventral sternum.

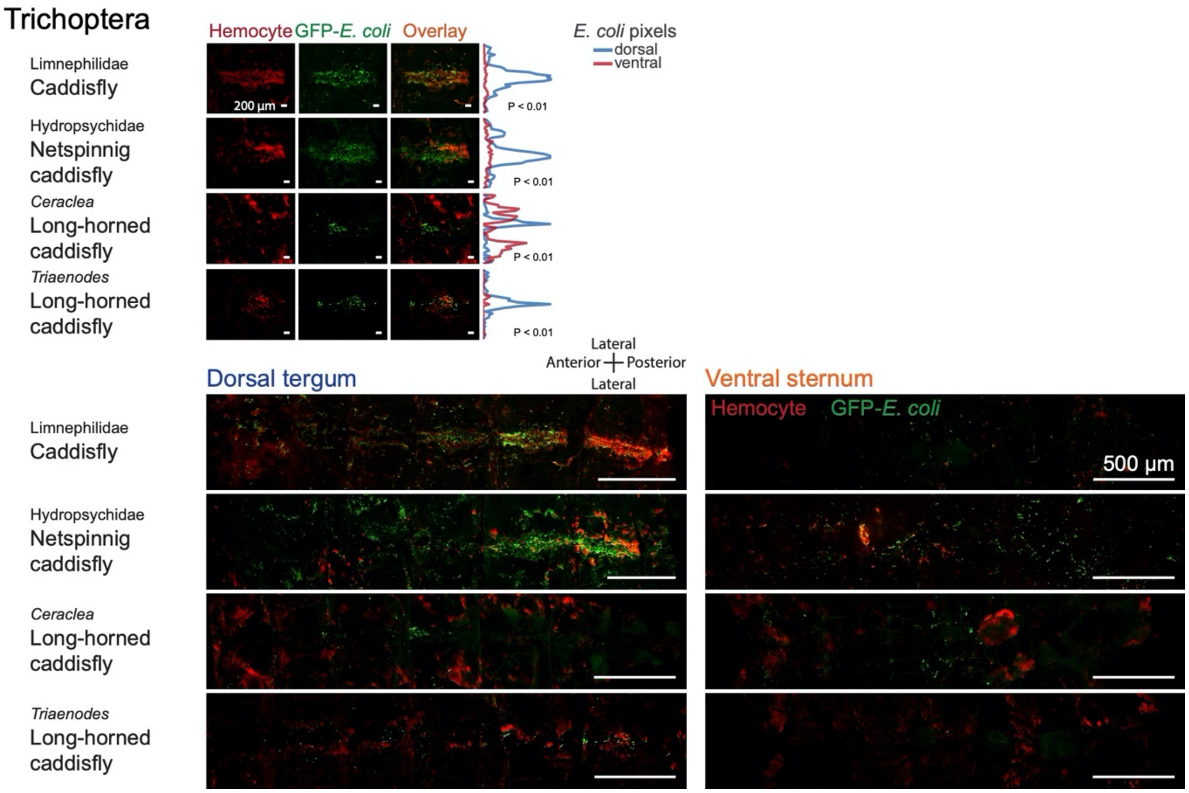

Fig. S5. The aggregation of hemocytes and pathogens on the heart of members of the order Trichoptera. Fluorescence microscopy images show one region of the heart (top section of the figure) and the entire dorsal and ventral abdomen (bottom section of the figure) in each insect. Graphs show quantification of GFP-*E. coli* pixel frequency along the width of the entire dorsal (blue line) and ventral (red line) abdomen. Hemocytes (red) and GFP-*E. coli* (green) distinctively aggregate and co-localize (overlay) on the heart – but not the surrounding tergum – and are largely absent in the ventral sternum.

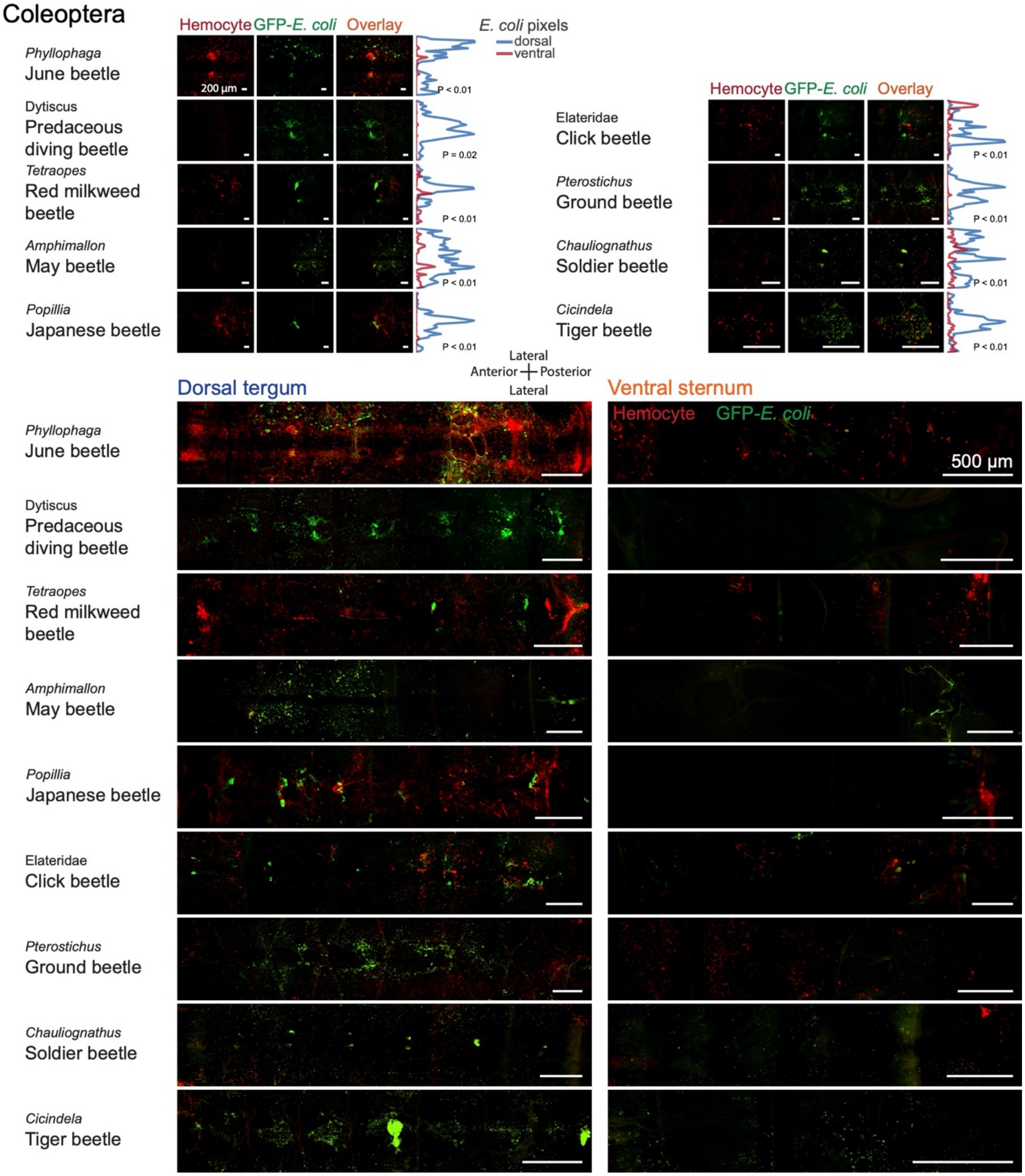

Fig. S6. The aggregation of hemocytes and pathogens on the heart of members of the order Coleoptera. Fluorescence microscopy images show one region of the heart (top section of the figure) and the entire dorsal and ventral abdomen (bottom section of the figure) in each insect. Graphs show quantification of GFP-*E. coli* pixel frequency along the width of the entire dorsal (blue line) and ventral (red line) abdomen. Hemocytes (red) and GFP-*E. coli* (green) distinctively aggregate and co-localize (overlay) on the heart – but not the surrounding tergum – and are largely absent in the ventral sternum.

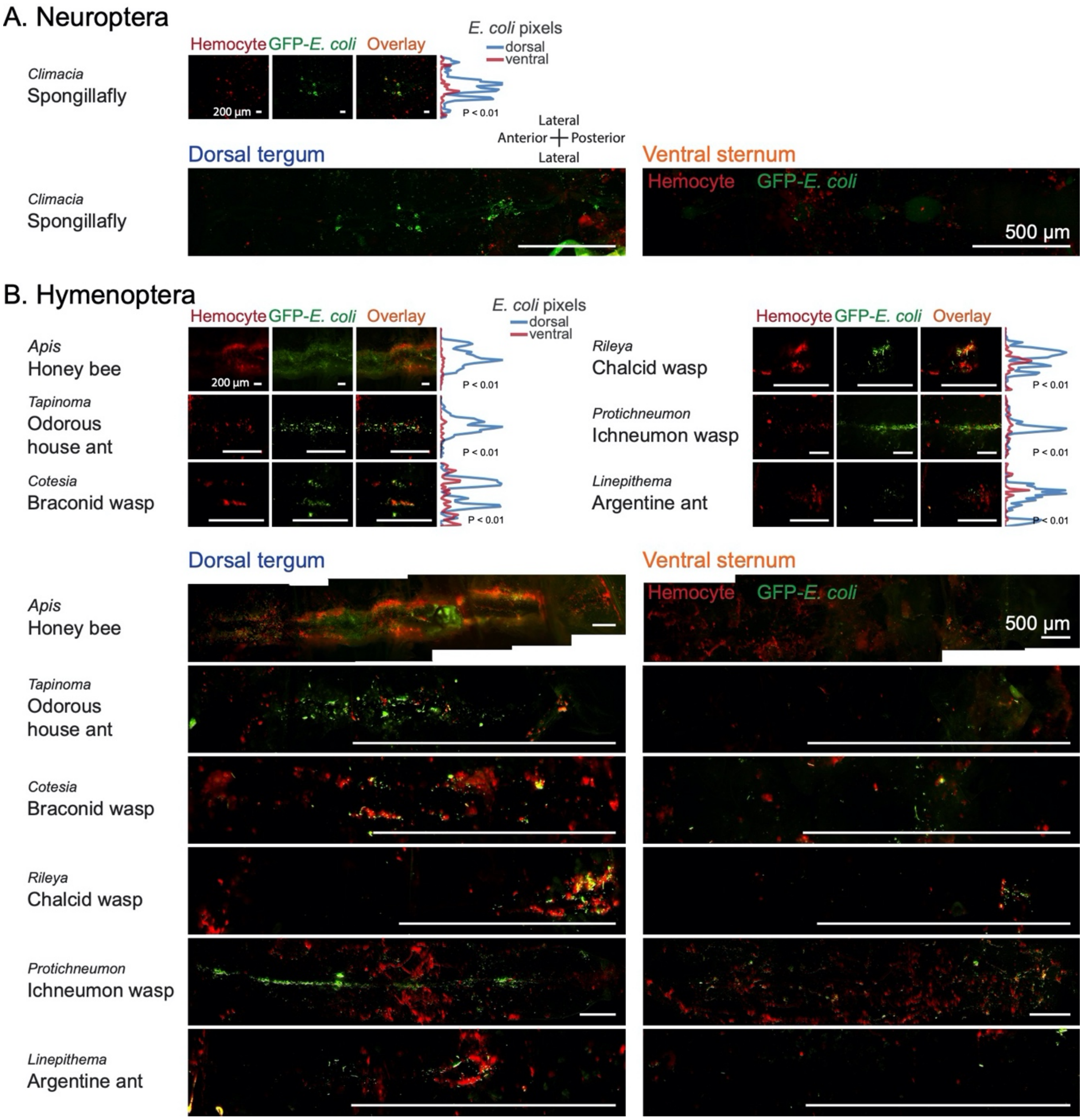

Fig. S7. The aggregation of hemocytes and pathogens on the heart of members of the orders Neuroptera and Hymenoptera. Fluorescence microscopy images show one region of the heart (top section of each panel) and the entire dorsal and ventral abdomen (bottom section of each panel) in each insect. Graphs show quantification of GFP-*E. coli* pixel frequency along the width of the entire dorsal (blue line) and ventral (red line) abdomen. In both Neuroptera (**A**) and Hymenoptera (**B**), hemocytes (red) and GFP-*E. coli* (green) distinctively aggregate and co-localize (overlay) on the heart – but not the surrounding tergum – and are largely absent in the ventral sternum.

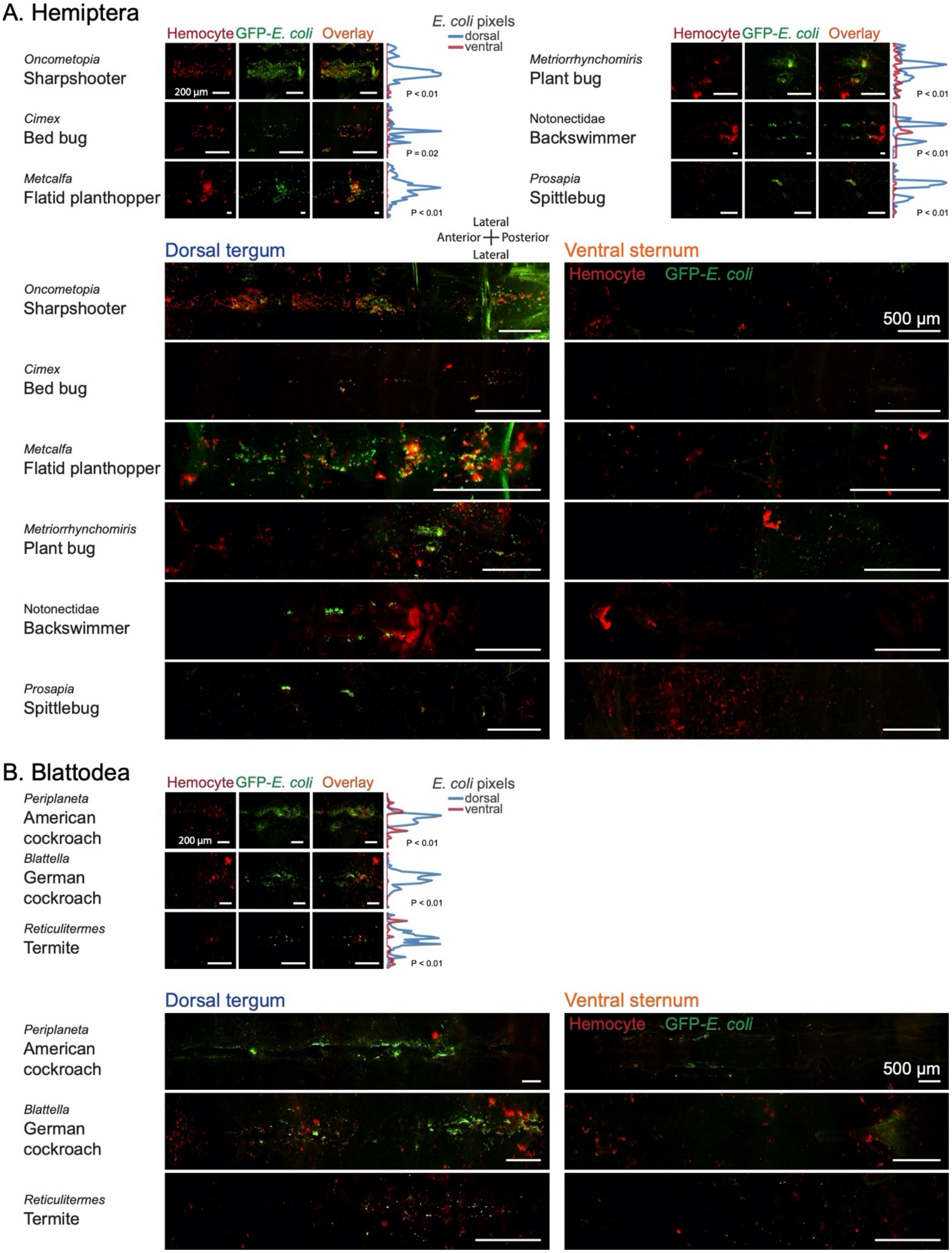

Fig. S8. The aggregation of hemocytes and pathogens on the heart of members of the orders Hemiptera and Blattodea. Fluorescence microscopy images show one region of the heart (top section of each panel) and the entire dorsal and ventral abdomen (bottom section of each panel) in each insect. Graphs show quantification of GFP-*E. coli* pixel frequency along the width of the entire dorsal (blue line) and ventral (red line) abdomen. In both Hemiptera (**A**) and Blattodea (**B**), hemocytes (red) and GFP-*E. coli* (green) distinctively aggregate and co-localize (overlay) on the heart – but not the surrounding tergum – and are largely absent in the ventral sternum.

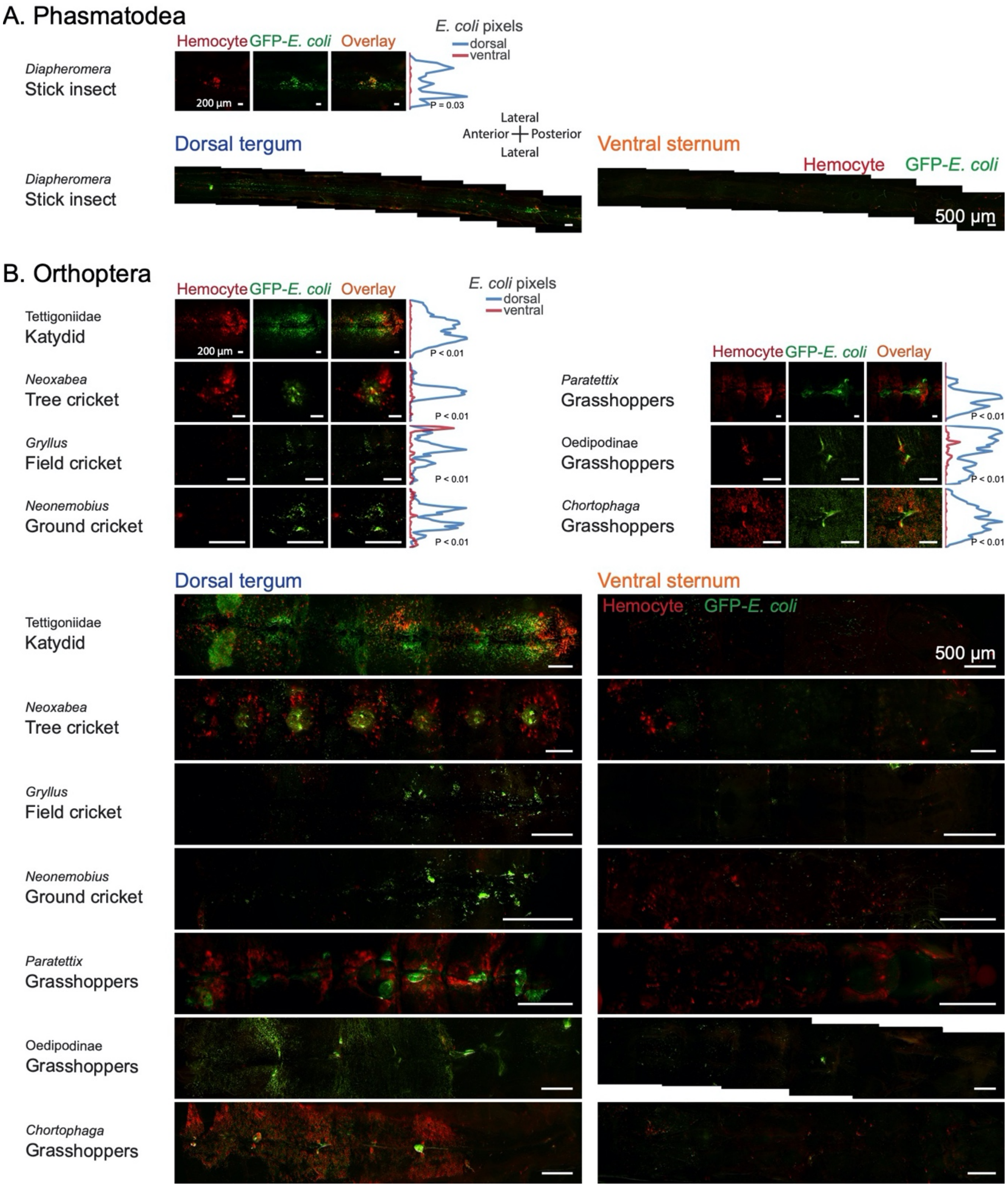

Fig. S9. The aggregation of hemocytes and pathogens on the heart of members of the orders Phasmatodea and Orthoptera. Fluorescence microscopy images show one region of the heart (top section of each panel) and the entire dorsal and ventral abdomen (bottom section of each panel) in each insect. Graphs show quantification of GFP-*E. coli* pixel frequency along the width of the entire dorsal (blue line) and ventral (red line) abdomen. In both Phasmatodea (**A**) and Orthoptera (**B**), hemocytes (red) and GFP-*E. coli* (green) distinctively aggregate and co-localize (overlay) on the heart – but not the surrounding tergum – and are largely absent in the ventral sternum.

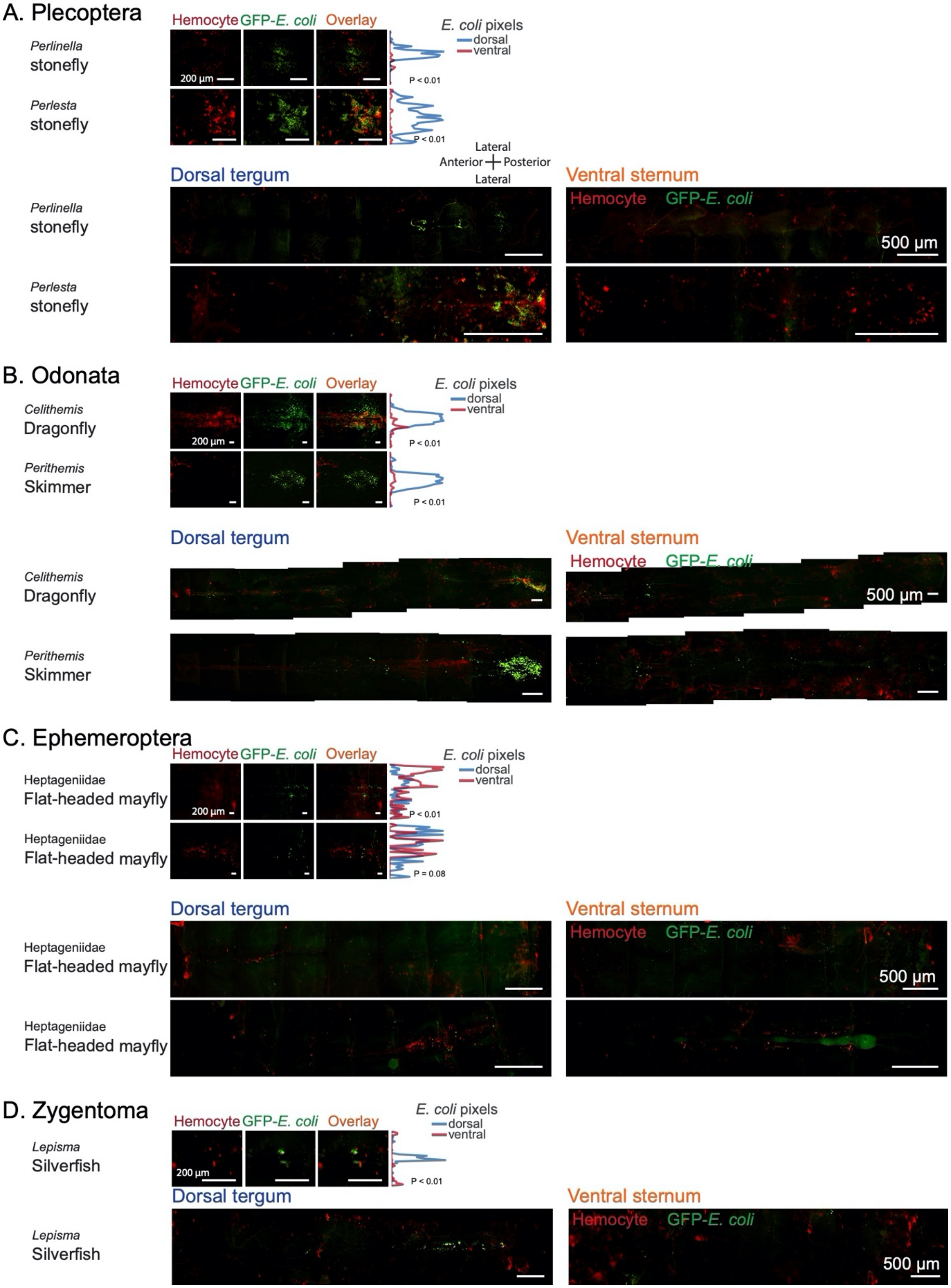

Fig. S10. The aggregation of hemocytes and pathogens on the heart of members of the orders Plecoptera, Odonata, Ephemeroptera and Zygentoma. Fluorescence microscopy images show one region of the heart (top section of each panel) and the entire dorsal and ventral abdomen (bottom section of each panel) in each insect. Graphs show quantification of GFP-*E. coli* pixel frequency along the width of the entire dorsal (blue line) and ventral (red line)

abdomen. In Plecoptera (**A**), Odonata (**B**) and Zygentoma (**D**), hemocytes (red) and GFP-*E. coli* (green) distinctively aggregate and co-localize (overlay) on the posterior end of the heart – but not the surrounding tergum – and are largely absent in the ventral sternum. In Ephemeroptera (**C**), hemocytes and GFP-*E. coli* do not aggregate or co-localize on the heart.

Table S1. Detailed information on the insects used in this study.

Order	Family	Species	Common name	Location collected	Date of collection	Collection method	Collector[a]	Volume Injected (nL)	Number of *E. coli* injected	Incubation after infection
Zygentoma	Lepismatidae	*Lepisma saccharina*	Silverfish	Mulberry way, Nashville, TN	3/21/2019	trap	YY	1497	330903	1 hour
Ephemeroptera	Heptageniidae	*Leucrocuta* sp.	Mayfly	Mulberry way, Nashville, TN	7/31/2019	light trap	YY	311	215798	1 hour
Ephemeroptera	Heptageniidae	Unidentified	Flat-headed mayfly	Mulberry way, Nashville, TN	6/24/2019	light trap	YY	200	106200	4 hours
Odonata	Libellulidae	*Celithemis elisa*	Dragonfly	Leopold, IN	7/28/2019	sweep net	YY	6141	4722429	1 hour
Odonata	Libellulidae	*Perithemis tenera*	Skimmer	Willow Pond, Nashville, TN	7/7/2018	sweep net	YY, LJ	4140	1349640	1 hour
Plecoptera	Perlidae	*Perlinella* sp.	Stonefly	Hidden Lake Trail, Harpeth River State Park, Nashville, TN	6/9/2018	sweep net	YY, LJ, JS, PR, MT	3795	2819685	1 hour
Plecoptera	Perlidae	*Perlesta* sp.	Stonefly	Bellevue, Nashville, TN	7/19/2019	light trap	LJ	759	53130	1 hour
Orthoptera	Tettigoniidae	Unidentified	Katydid	Mulberry way, Nashville, TN	7/30/2019	light trap	YY	20100	10311300	1 hour
Orthoptera	Gryllidae	*Neoxabea* sp.	Tree cricket	Mulberry way, Nashville, TN	7/21/2019	sweep net	YY	4830	1197840	1 hour
Orthoptera	Gryllidae	*Gryllus* sp.	Field cricket	Deer Trail, Long Hunter State Park, TN	4/28/2019	sweep net	YY, LJ, PR, EH, MW	15000	14520000	4 hours
Orthoptera	Gryllidae	*Neonemobius* sp.	Ground cricket	Willow Pond, Nashville, TN	7/7/2018	sweep net	YY, LJ	3126	3344499	1 hour
Orthoptera	Tetrigidae	*Paratettix* sp.	Grasshopper	Willow Pond, Nashville, TN	5/25/2019	sweep net	YY, LJ, JC, DG	3933	3028410	4 hours
Orthoptera	Acrididae	Unidentified	Grasshopper	Deer Trail, Long Hunter State Park, TN	4/28/2019	sweep net	YY, LJ, PR, EH, MW	30000	29460000	4 hours
Orthoptera	Acrididae	*Chortophaga* sp.	Grasshopper	Hidden Lake Trail, Harpeth River State Park, Nashville, TN	6/9/2018	sweep net	YY, LJ, JS, PR, MT	17733	13175619	1 hour
Phasmatodea	Diapheromeridae	*Diapheromera femorata*	Walking stick	Bellevue, Nashville, TN	8/21/2019	sweep net	MGG	15870	6824100	1 hour
Blattodea	Blattidae	*Periplaneta americana*	American cockroach	Purdue University, West Lafayette, IN	5/6/2019	lab colony	GB	43263	29245788	4 hours
Blattodea	Ectobiidae	*Blattella germanica*	German cockroach	Purdue University, West Lafayette, IN	5/6/2019	lab colony	GB	5590	3778840	4 hours
Blattodea	Rhinotermitidae	*Reticulitermes flavipes*	Termite	Purdue University, West Lafayette, IN	5/6/2019	lab colony	GB	69	66171	4 hours
Hemiptera	Cicadellidae	*Oncometopia* sp.	Sharpshooter	Mulberry way, Nashville, TN, USA	5/24/2018	sweep net	YY	3588	2619240	1 hour
Hemiptera	Cimicidae	*Cimex lectularius*	Bed bug	Purdue University, West Lafayette, IN	5/6/2019	lab colony	GB, AG	276	186576	4 hours
Hemiptera	Flatidae	*Metcalfa* sp.	Flatid planthopper	Mulberry way, Nashville, TN	8/4/2019	light trap	YY	725	607131	1 hour
Hemiptera	Miridae	*Metriorrhynchomiris* sp.	Plant bug	Gossett Tract, Harpeth River State Park, Kingston Springs, TN	5/18/2019	sweep net	YY, SW, MT, MGG	828	355212	4 hours
Hemiptera	Notonectidae	Unidentified	Backswimmer	West Lafayette, IN	6/29/2019	light trap	SW	1380	1188180	4 hours
Hemiptera	Cercopidae	*Prosapia bicincta*	Spittlebug	Willow Pond, Nashville, TN	7/7/2018	sweep net	YY, LJ	2760	2969760	1 hour
Hymenoptera	Apidae	*Apis mellifera*	Honeybee	Vanderbilt University Campus, Nashville, TN	5/23/2018	sweep net	LJ	18561	13549530	1 hour

Hymenoptera	Formicidae	*Tapinoma sessile*	Odorous house ant	Purdue University, West Lafayette, IN	5/6/2019	lab colony	GB	40	37360	4 hours
Hymenoptera	Braconidae	*Cotesia* sp.	Braconid wasp	Nolensville pike, TN (emerged from hornworm caterpillar)	7/18/2019	by hand	PR	46	9062	1 hour
Hymenoptera	Eurytomidae	*Rileya* sp.	Chalcid wasp	Willow Pond, Nashville, TN	5/25/2019	sweep net	YY, LJ, JC, DG	27	20790	4 hours
Hymenoptera	Ichneumonidae	*Protichneumon grandis*	Ichneumon wasp	Willow Pond, Nashville, TN	7/7/2018	sweep net	YY, LJ	4009	3700215	1 hour
Hymenoptera	Formicidae	*Linepithema humile*	Argentine ant	Purdue University, West Lafayette, IN	5/6/2019	lab colony	GB	40	37360	4 hours
Neuroptera	Sisyridae	*Climacia* sp.	Spongillafly	Mulberry way, Nashville, TN	7/9/2019	light trap	YY	173	132825	1 hour
Coleoptera	Scarabaeidae	*Phyllophaga* sp.	June beetle	Mulberry way, Nashville, TN	6/18/2019	light trap	YY	11730	3190560	4 hours
Coleoptera	Dytiscus	Unidentified	Predaceous diving beetle	West Lafayette, IN	7/15/2019	light trap	SW	4913	4116926	1 hour
Coleoptera	Cerambycidae	*Tetraopes* sp.	Red milkweed beetle	West Lafayette, IN	7/21/2019	sweep net	SW	8280	6375600	1 hour
Coleoptera	Scarabaeidae	*Amphimallon* sp.	May beetle	Bellevue, Nashville, TN	4/21/2019	light trap	JH	9522	9998100	1 hour
Coleoptera	Scarabaeidae	*Popillia japonica*	Japanese beetle	Vanderbilt University Campus, Nashville, TN	6/17/2019	sweep net	YY	6486	252954	4 hours
Coleoptera	Elateridae	Unidentified	Click beetle	Mulberry way, Nashville, TN	6/18/2019	light trap	YY	5106	3497610	4 hours
Coleoptera	Carabidae	*Pterostichus* sp.	Ground beetle	Bellevue, Nashville, TN	6/8/2019	trap	LJ	15870	11934240	1 hour
Coleoptera	Cantharidae	*Chauliognathus marginatus*	Soldier beetle	Hidden Lake Trail, Harpeth River State Park, Nashville, TN	6/2/2018	sweep net	YY, LJ, JC, JS	3243	1563126	1 hour
Coleoptera	Carabidae	*Cicindela sexguttata*	Tiger beetle	Hidden Lake Trail, Harpeth River State Park, Nashville, TN	6/9/2018	sweep net	YY, LJ, JS, PR, MT	5382	3998826	1 hour
Trichoptera	Limnephilidae	Unidentified	Northern caddisfly	Mulberry way, Nashville, TN	7/28/2019	light trap	YY	276	212244	1 hour
Trichoptera	Hydropsychidae	Unidentified	Net-spinning caddisfly	Mulberry way, Nashville, TN	7/31/2019	light trap	YY	1297	901554	1 hour
Trichoptera	Leptoceridae	*Ceraclea* sp.	Long-horned caddisfly	Mulberry way, Nashville, TN	7/29/2019	light trap	YY	173	127995	1 hour
Trichoptera	Leptoceridae	*Triaenodes* sp.	Long-horned caddisfly	Mulberry way, Nashville, TN	7/10/2019	light trap	YY	235	66157	1 hour
Lepidoptera	Noctuidae	*Mythimna* sp.	Armyworm moth	Mulberry way, Nashville, TN	6/18/2019	light trap	YY	14490	3941280	4 hours
Lepidoptera	Noctuidae	*Striacosta albicosta*	Western bean cutworm	Cornfield, IN	7/11/2019	sweep net	SW	10557	2079729	1 hour
Lepidoptera	Erebidae	*Spilosoma* sp.	Tiger moth	Willow Pond, Nashville, TN	5/25/2019	sweep net	YY, LJ, JC, DG	5589	4303530	4 hours
Lepidoptera	Geometridae	Unidentified	Geometer moth	Deer Trail, Long Hunter State Park, Hermitage, TN	4/28/2019	sweep net	YY, LJ, PR, EH, MW	759	745338	4 hours
Lepidoptera	Crambidae	*Desmia* sp.	Grape leaf folder	Willow Pond, Nashville, TN	7/7/2018	sweep net	YY, LJ	2491	2680208	1 hour
Lepidoptera	Pyralidae	*Plodia interpunctella*	Indian meal moth	Purdue University, West Lafayette, IN	5/6/2019	lab colony	SW	552	395232	1 hour
Lepidoptera	Erebidae	*Renia* sp.	Litter moth	Gossett Tract, Harpeth River State Park, Kingston Springs, TN	5/18/2019	sweep net	YY, SW, MT, MGG	2484	1967328	4 hours
Lepidoptera	Nymphalidae	*Hermeuptychia* sp.	Satyrid butterfly	Willow Pond, Nashville, TN	7/1/2018	sweep net	YY, LJ	2491	3437442	1 hour
Lepidoptera	Nymphalidae	*Danaus plexippus*	Monarch butterfly	Leopold, IN	7/28/2019	sweep net	YY	45512	33769904	1 hour
Lepidoptera	Lycaenidae	*Calycopis* sp.	Red-banded hairstreak	Willow Pond, Nashville, TN	7/1/2018	sweep net	YY, LJ	1270	1752048	1 hour

Lepidoptera	Nymphalidae	*Chlosyne* sp.	Checkerspot	Willow Pond, Nashville, TN	7/1/2018	sweep net	YY, LJ	1608	2218626	1 hour
Mecoptera	Panorpidae	*Panorpa* sp.	Scorpionfly	Gossett Tract, Harpeth River State Park, Kingston Springs, TN	5/18/2019	sweep net	YY, SW, MT, MGG	2484	1967328	4 hours
Mecoptera	Bittacidae	*Bittacus* sp.	Hangingfly	Willow Pond, Nashville, TN	7/7/2018	sweep net	YY, LJ	1546	1545600	1 hour
Siphonaptera	Pulicidae	*Ctenocephalides felis*	Cat flea	Bellevue, Nashville, TN	7/13/2019	trap	LJ	69	18768	1 hour
Diptera	Culicidae	*Culex* sp.	Mosquito	Cheekwood Garden, Nashville, TN	5/17/2019	scooping	YY	207	80523	1 hour
Diptera	Culicidae	*Aedes albopictus*	Mosquito	Vanderbilt University Campus, Nashville, TN	7/21/2019	sweep net	YY, LJ	90	22246	1 hour
Diptera	Culicidae	*Anopheles punctipennis*	Mosquito	Mulberry way, Nashville, TN	8/15/2019	trap	YY	104	67068	1 hour
Diptera	Muscidae	*Musca domestica*	House fly	Belle Forest Cave, Bellevue, TN	5/12/2018	sweep net	YY, JS	235	142061	1 hour
Diptera	Tephritidae	*Procecidochares* sp.	Fruit fly	Willow Pond, Nashville, TN	5/25/2019	sweep net	YY, LJ, JC, DG	414	318780	4 hours
Diptera	Mycetophilidae	*Leia bivittate*	Fungus gnat	Hidden Lake Trail, Harpeth River State Park, Nashville, TN	6/9/2018	sweep net	YY, LJ, JS, PR, MT	173	129720	1 hour
Diptera	Sarcophagidae	*Sarcophaga* sp.	Flesh fly	Gossett Tract, Harpeth River State Park, Kingston Springs, TN	5/18/2019	sweep net	YY, SW, MT, MGG	1104	473616	4 hours
Diptera	Tachinidae	Unidentified	Tachinid fly	North Judson, IN (emerged from monarch caterpillar)	8/4/2019	by hand	SW	1201	1006103	1 hour
Diptera	Dolichopodidae	*Dolichopus* sp.	Long-legged fly	Hidden Lake Trail, Harpeth River State Park, Nashville, TN	6/9/2018	sweep net	YY, LJ, JS, PR, MT	676	508502	1 hour
Diptera	Tabanidae	*Tabanus* sp.	Striped horse fly	Leopold, IN	7/28/2019	sweep net	YY	2001	1538769	1 hour

[a] YY = Yan Yan; LJ = Luisa Jabbur; JS = Jacob Steenwyk; PR = Parker Rundstrom; MT = Michael Tackenberg; EH = Emily Hudson; MW = Matt Wilkins; JC = Justin Critchlow; DG = Destane Garrett; MGG = Manuel Giannoni Guzman; GB = Grzegorz Buczkowski; AG = Ameya Gondhalekar; SW = Scott Williams; JH = Julián Hillyer.
